# Augmented BMPRIA-Mediated BMP Signaling in Cranial Neural Crest Lineage Leads to Cleft Palate Formation and Delayed Tooth Differentiation

**DOI:** 10.1371/journal.pone.0066107

**Published:** 2013-06-12

**Authors:** Lu Li, Ying Wang, Minkui Lin, Guohua Yuan, Guobin Yang, Yuqian Zheng, YiPing Chen

**Affiliations:** 1 Department of Cell and Molecular Biology, Tulane University, New Orleans, Louisiana, United States of America; 2 Department of Operative Dentistry and Endodontics, College of Stomatology, The Fourth Military Medical University, Xi'an, Shaanxi Province, P.R. China; 3 Department of Periodontology, College of Stomatology, Fujian Medical University, Fuzhou, Fujian Province, P.R. China; 4 Department of Pediatric Dentistry, College of Stomatology, Wuhan University, Wuhan, Hubei Province, P.R. China; Instituto de Medicina Molecular, Portugal

## Abstract

The importance of BMP receptor Ia (BMPRIa) mediated signaling in the development of craniofacial organs, including the tooth and palate, has been well illuminated in several mouse models of loss of function, and by its mutations associated with juvenile polyposis syndrome and facial defects in humans. In this study, we took a gain-of-function approach to further address the role of BMPR-IA-mediated signaling in the mesenchymal compartment during tooth and palate development. We generated transgenic mice expressing a constitutively active form of *BmprIa* (*caBmprIa*) in cranial neural crest (CNC) cells that contributes to the dental and palatal mesenchyme. Mice bearing enhanced BMPRIa-mediated signaling in CNC cells exhibit complete cleft palate and delayed odontogenic differentiation. We showed that the cleft palate defect in the transgenic animals is attributed to an altered cell proliferation rate in the anterior palatal mesenchyme and to the delayed palatal elevation in the posterior portion associated with ectopic cartilage formation. Despite enhanced activity of BMP signaling in the dental mesenchyme, tooth development and patterning in transgenic mice appeared normal except delayed odontogenic differentiation. These data support the hypothesis that a finely tuned level of BMPRIa-mediated signaling is essential for normal palate and tooth development.

## Introduction

Bone morphogenetic protein (BMP) signaling plays pivotal roles in development of almost every organ during embryogenesis. BMP signaling is transduced into cells by binding of ligands to the type I and type II transmembrane serine/threonine kinase complex. Upon binding of ligand, the type II receptor activates the type I receptor by phosphorylating the type I receptor, the latter further phosphorylates the receptor-regulated Smad (rSmad), primarily Smad-1, -5, and -8, in the cytoplasm. Phosphorylated rSmads bind to common Smad (Smad4) and enter the nucleus to regulate gene expression. In addition to this canonical (Smad-dependent) pathway, ligand-occupied BMP receptor complex can also activate directly Smad-independent (non-canonical) pathways resulting in activation of the mitogen-activated protein kinase signaling [1]. There are two primary type I BMP receptors in vertebrates, BMPRIa and BMPRIb. Mice with *BmprIb* deficiency are viable with limb defects [2], [3], but inactivation of *BmprIa* leads to embryonic lethality at early gestation stage [4], indicating a profound role for BMPRIa-mediated signaling in embryonic development.

The cranial neural crest (CNC) cells contribute to various types of tissues of developing craniofacial organs, including the dental mesenchyme and palatal mesenchyme. The development of tooth and palate required a series of interactions between pharyngeal ectoderm and CNC-derived mesenchyme. It has been well documented that these interactions are mediated by multiple families of growth factors including BMP [5], [6]. In the developing palate, several *Bmp* genes are expressed in dynamic and differential patterns along the anterior-posterior (A-P) axis [7], [8], and BMP signaling has been shown to regulate cell proliferation in the anterior palatal mesenchyme and to maintain palatal epithelial integrity in the posterior portion [5], [9], [10], [11], [12], [13]. In the developing tooth, BMP signaling has been implicated in almost every step of odontogenesis, including determination of tooth-forming site and tooth type [14], [15], initiation [16], progression from the bud to cap stage and enamel knot formation [17], [18], [19], [20], as well as tooth root formation and tooth eruption [21], [22], [23], [24].

Loss-of-function studies have pinpointed to the central importance of BMPRIa in mediating BMP signaling during palate and tooth development. Inactivation of *BmprIa* in the maxillary mesenchyme and oral epithelium led to cleft lip and palate [25]. Despite normal palate formation, disruption of *BmprIa* in the epithelium caused an arrest of tooth development at the bud/cap stage [26]. Tissue-specific inactivation of *BmprIa* in CNC lineage or in the palatal mesenchyme resulted in anterior clefting of the secondary palate attributed to a decreased cell proliferation rate in the anterior palatal mesenchyme [12], [13]. *BmprIa* deficiency in CNC lineage also arrested tooth development at the bud/cap stage associated with decreased levels of cell proliferation and down-regulation of several BMP downstream genes in the dental mesenchyme [13]. Interestingly, in a dominant-negative transgenic mouse model, it was shown that reduced BMPRIa-mediated signaling caused facial dysmorphism and cleft palate, mimicking the hypertelorism and flat nasal bridge observed in patients with juvenile polyposis syndrome and chromosome 10q23 deletion syndrome that are associated with *BMPRIA* mutations or deletion [27], [28], [29], [30], [31]. The indispensable role of *BmprIa* is further supported by the fact that *BmprIb* has limited redundant function with *BmprIa* in tooth and palate development [13].

We have reported previously that ectopic transgenic expression of a constitutively active form of *BmprIa* (*caBmprIa*) in the palatal epithelium resulted in abnormal fusion of the developing palate with the mandible and subsequently the cleft palate formation, resembling the palate defect observed in mice lacking the BMP antagonist Noggin [11]. To further investigate the role of BMPRIa-mediated signaling in the mesenchymal compartment during palate and tooth development, we expressed *caBmprIa* in the CNC lineage. We showed that enhanced BMPRIa-mediated signaling in CNC-derived palatal and dental mesenchyme leads to complete clefting of the secondary palate and delayed odontogenic differentiation, further supporting the hypothesis that a finely tuned level of BMP signaling is essential for normal palate and tooth development.

## Materials and Methods

### Animals

Generation of the conditional transgenic mice expressing a constitutively active form (with Gln203 to Asp change) of *BmprIa* (*pMes*-*caBmprIa*) has been described previously [11]. *Wnt1*-*Cre* mice [32] were obtained from Jackson Laboratories. *Wnt1*-*Cre* mice were mated to *pMes*-*caBmprIa* mice to obtain *Wnt1*-*Cre*;*pMes*-*caBmprIa* mice. Binary transgenic embryos were harvested from timed pregnant females, and tail sample from each embryo was subjected to PCR-based genotyping.

### Ethics statement

Use of animals in this study was approved by the Institutional Animal Care and Use Committee (IACUC) of Tulane University (protocol number: 0329R2) and was in strict accordance with the recommendations in the Guide for Care and Use of Laboratory Animals of the National Institutes of Health.

### Histology, in situ hybridization, immunohistochemistry, BrdU labeling, and subrenal culture

For histology and section in situ hybridization analyses, staged embryonic heads were fixed in 4% paraformaldehyde (PFA) at 4°C overnight and then processed for paraffin section at 10-µm. Standard hematoxylin/Eosin staining and non-radioactive in situ hybridization were performed as described previously [33]. For immunohistochemical staining, embryonic heads were fixed in 4% PFA at 4°C for 2 hr, embedded in O.C.T. (Tissue-Tek), and cryo-sectioned at 10-µm. Immunohistochemical staining using antibodies against pSmad1/5/8 (from Cell Signaling, cat #: 9511), pSmad2/3 (Santa Cruz, cat #: sc-11769), P-p38 (R&D, cat #: AF869), P-Erk (R&D, cat #: AF1018), and P-JNK (R&D, cat #: AF1205) was conducted as described previously [11]. BrdU labeling was conducted to determine cell proliferation rate as described previously [9]. Briefly timed pregnant female mice were injected intraperitoneally with BrdU solution (1.5 ml/100 g body weight) from the BrdU Labeling and Detection Kit (Roche) 1 hr prior to embryo harvest. Embryonic heads were fixed in Carnoy's fixative, paraffin-embedded, and sectioned at 5-µm. Sections were subjected to immunostaining according to the manufacturer's instruction. Cell proliferation rates were measured by counting BrdU-positive cells and total cells within defined arbitrary areas, and presented as percentage of labeled cells against total cells in the fixed area. Three control and three transgenic embryos were used for BrdU labeling study. Data were collected from three continuous sections from each embryo and the sums from both genotypes were subjected to Student's *t*-test to determine the significance of difference. For kidney capsule grafting, the first mandibular molars were isolated from postnatal day 0 (P0) wild type and transgenic mice and subjected to subrenal culture in adult CD-1 male mice as described previously [34]. Samples were retrieved 2 weeks after subrenal culture, decalcified, and processed for histology and in situ hybridization.

## Results

### Expression of caBmprIa in CNC-derived tissues causes palate cleft

**Figure 1:**
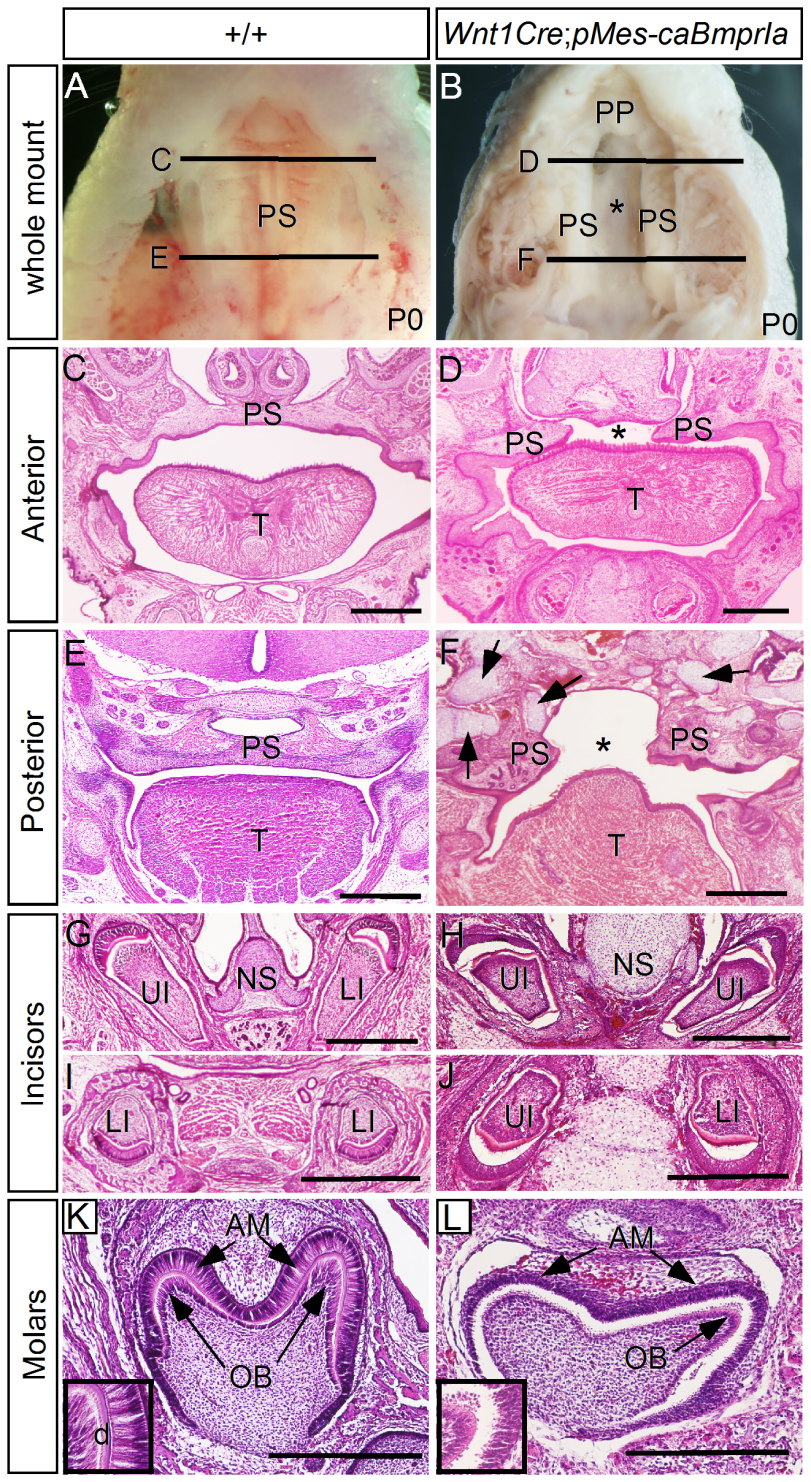
Enhanced BMP activity in CNC-derived tissues via caBMPRIa causes complete cleft palate. (A, C, E) Whole mount and coronal sections show normal palatal shelf of P0 wild type mice. Black lines in (A) indicate section levels shown in (C) and (E). (B, D, F) Whole mount and coronal sections show complete cleft (denoted by asterisk) of the secondary palate of P0 *Wnt1Cre*;*pMes*-*caBmprIa* mice. Note presence of ectopic cartilages (arrows) in craniofacial region. Black lines in (B) indicate section levels shown in (D) and (F). (G–J) Coronal sections of P0 control and *Wnt1Cre*;*pMes*-*caBmprIa* mice show comparable morphology of upper and lower incisors. Note enlarged nasal septal cartilage in transgenic animal. (K, L) Coronal sections of P0 control and transgenic mice show first molar structure with less differentiated odontoblasts and ameloblasts (inserts) in transgenic animal. T, tongue; AM, ameloblasts; LI, lower incisor; NS, nasal septum; OB, odontoblasts; PS, palatal shelf; UI, upper incisor. Scale bar = 500 µm.

To elevate BMPRIa-mediated signaling in the palatal and dental mesenchyme, we bred *Wnt1*-*Cre* mice with *pMes*-*caBmprIa* mice to generate *Wnt1*-*Cre*;*pMes*-*caBmprIa* mice. The function of the *pMes*-*caBmprIa* transgenic allele has been demonstrated previously [11]. All binary transgenic mice died shortly after birth. Gross morphological examination revealed a cleft palate defect (Fig. 1A, 1B). Among 16 *Wnt1Cre;pMes-caBmprIa* mice that were examined, all of them had complete cleft of the secondary palate, with two being accompanied with unilateral cleft lip, and one with bilateral cleft lip (data not shown). However, the primary palate appeared normal (Fig. 1B). Histological analysis of postnatal day 0 (P0) transgenic animals revealed that the palatal shelves were elevated to the position above the tongue in both the anterior and posterior region, but failed to meet at the midline (Fig. 1C–F). While the transgenic incisors exhibited structures including dentin deposition morphologically comparable to controls (Fig. 1G–J), the transgenic molars showed less differentiated (shortened) odontoblasts and ameloblasts and lacked dentin deposition (Fig. 1K, 1L, and inserts), despite normal size and cusp patterns (see below). In addition, ectopic cartilages and enlarged nasal septal cartilage were present in the craniofacial region of *Wnt1Cre;pMes-caBmprIa* mice (Fig. 1F, 1H).

### Augmented BMP signaling leads to deformed palate structure and delayed palatal elevation

**Figure 2:**
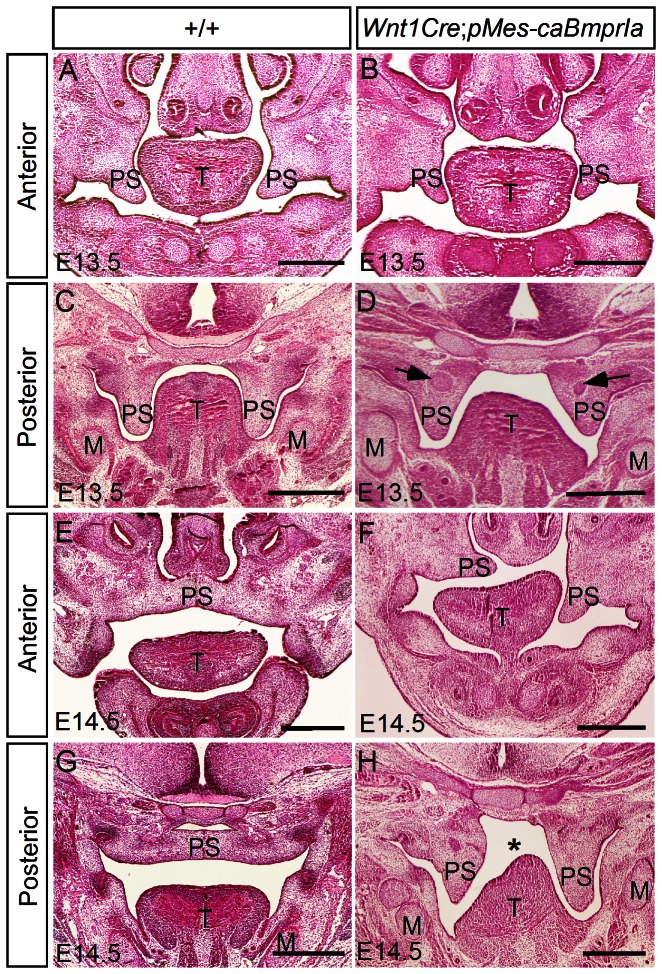
Deformed structure and delayed elevation of palatal shelves in *Wnt1Cre*;*capMes*-*caBmprIa* mice. (A–C) Coronal sections of E13.5 control and *Wnt1Cre*;*pMes*-*caBmprIa* embryos show deformed morphology of palatal shelves in transgenic animals. Note the presence of ectopic condensed cell masses (arrows) within in the posterior palatal shelves of the transgenic embryo (Fig. 2D). (E–H) Coronal sections of E14.5 wild type and *Wnt1Cre*;*pMes*-*caBmprIa* embryos show delayed elevation of palatal shelves in transgenic animal. M, Meckel's cartilage; T, tongue; PS, palatal shelf. Scale bar = 500 µm.

In order to reveal cellular and molecule bases underlying the cleft palate phenotype observed in *Wnt1Cre*;*caBmprIa* mice, we first analyzed palatogenetic process in transgenic embryos. At E11.5 and E12.5, the palatal shelves of transgenic animals exhibited morphologically comparable structures to the controls (data not shown). At E13.5, although the transgenic palatal shelves took a vertical position at both sides of the developing tongue along the anterior-posterior axis, similar to that in the wild type controls, the transgenic palatal shelves appeared smaller in size in the anterior portion and were shortened and much wider in the posterior portion (Fig. 2A–D). In addition, an ectopic condensed mesenchymal cell mass formed in the middle region of each palatal shelf in the posterior domain (Fig. 2D). At E14.5 when the palatal shelves in wild type control have elevated to the position above the tongue and have met at the midline, the transgenic palatal shelves were either not elevated or sometimes elevated on one side (Fig. 2E–H). Thus overexpression of *caBmprIa* in CNC-derived palatal mesenchyme causes a defective development of palatal shelves, and ultimately leads to the formation of complete cleft of the secondary palate.

**Figure 3:**
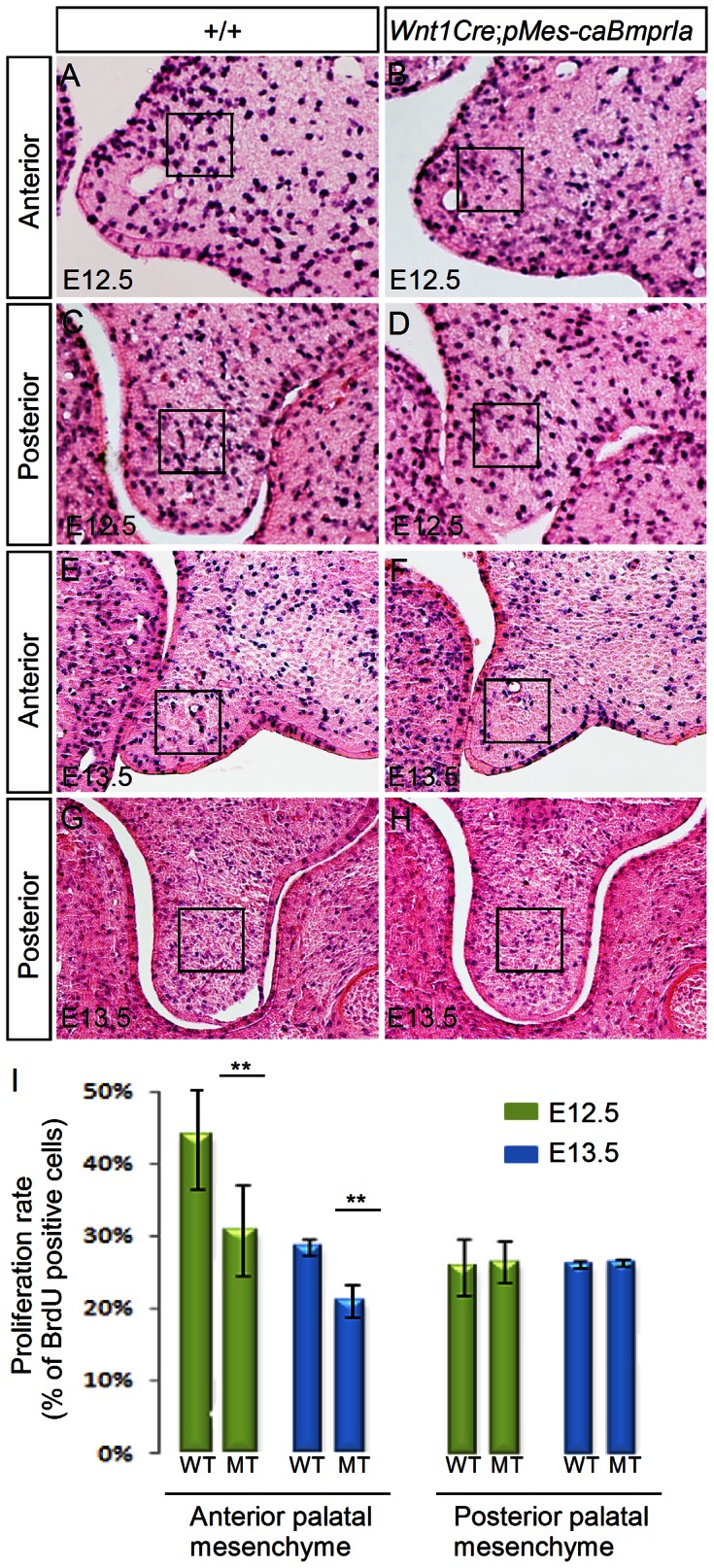
Reduced cell proliferation rate in the anterior palatal mesenchyme of *Wnt1Cre*;*pMes*-*caBmprIa* embryo. (A–H) Coronal sections show BrdU-labeled cells in the palatal shelves of E12.5 (A–D) and E13.5 (E–H) control and *Wnt1Cre*;*pMes*-*caBmprIa* embryos. Square box in each panel indicates the area where total cells and BrdU-positive cells were counted. (I) Comparison of percentage of BrdU-labeled cells in the designated area of the palatal shelves in the control and transgenic animals. Standard deviation values were presented as error bars, and ** indicates *P*<0.01.

To investigate cellular defects that may contribute to a cleft palate formation in *Wnt1Cre*;*pMes-caBmprIa* embryos, we carried out BrdU labeling and TUNEL assays to examine cell proliferation rates and apoptosis. In the developing palatal shelves of the transgenic embryo at E12.5 and E13.5, we detected a significantly reduced level of cell proliferation in the mesenchyme of the anterior palate, as compared to that in the controls (Fig. 3). However, cell proliferation rates in the posterior palatal mesenchyme remained unchanged (Fig. 3) (N = 3 for each genotype at each time point). On the other hand, TUNEL assays did not reveal enhanced/ectopic cell apoptosis in the palatal shelves of the transgenic animals at these stages (data not shown). Thus this reduced cell proliferation rate in the mesenchymal compartment represents one defective cellular mechanism contributing to a cleft palate formation in *Wnt1Cre*;*pMes-caBmprIa* mutants.

### Altered gene expression pattern associated with ectopic cartilage formation in the posterior palatal shelves of Wnt1Cre;pMes-caBmprIa mice

**Figure 4:**
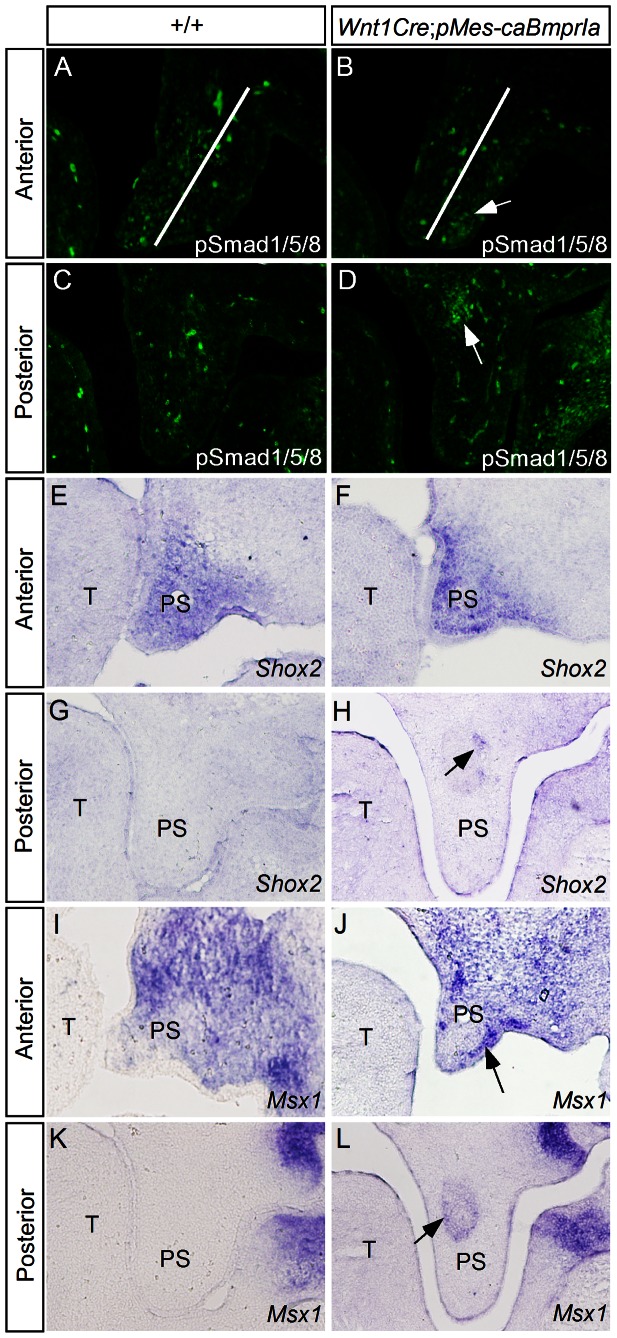
Altered BMP/Smad signaling activity and gene expression in *Wnt1Cre*;*pMes* **-** *caBmprIa* palatal shelves. (A–D) Immunostaining shows pSmad1/5/8 signals in the palatal mesenchyme of E13.5 wild type (A, C) and transgenic embryos (B, C). Note in the anterior palatal shelf, pSmad1/5/8 signals were shifted to the future oral side (arrow) in the anterior palatal mesenchyme (B) and were ectopically activated (arrow) in the posterior palatal mesenchyme (D) of the transgenic palatal shelves. (E–H) In situ hybridization shows unaltered *Shox2* expression in the anterior palatal mesenchyme (F) but an ectopic *Shox2* expression domain (arrow) in the posterior palatal shelf (H) of E13.5 *Wnt1Cre*;*pMes*-*caBmprIa* embryo as compared to the counterpart of controls (E, G). (I–L) In situ hybridization shows a strong *Msx1* expression domain (arrow) in the oral side of anterior palatal mesenchyme (J) and an ectopic *Msx1* expression domain in the posterior palatal shelf (L) of E13.5 *Wnt1Cre*;*pMes*-*caBmprIa* embryo as compared to the controls (I, K). T, tongue; PS, palatal shelf.

To determine how expression of *caBmprIa* in the CNC lineage alters BMP signaling in the palatal mesenchyme, we examined the expression of phosphorylated Smad1/5/8 (pSmad1/5/8) by immunohistochemical staining. In the wild type controls at E13.5, we detected pSmad1/5/8 positive cells primarily in the anterior palatal mesenchyme primarily in the future nasal side, and sporadic pSmad1/5/8 positive cells in the posterior palatal mesenchyme (Fig. 4A, 4C). Interestingly in the transgenic palatal shelves, we did not observed significantly increased number of pSmad1/5/8 positive cells, but found shift of pSmad1/5/8 positive cells to the future oral side in the anterior palatal mesenchyme and an ectopic mass of pSmad1/5/8 positive cells in the posterior palatal mesenchyme (Fig. 4B, D).

Msx1 and Shox2 transcription factors, the downstream targets of BMP signaling, are expressed in the anterior palatal mesenchyme and play critical roles in palate development [9], [13], [35]. We performed in situ hybridization to examine if altered BMP signaling in the palatal mesenchyme would affect the expression of these two genes. In the anterior palate of transgenic embryos at E13.5, *Shox2* expression remained unchanged compared to the control, but enhanced *Msx1* expression was observed in the future oral side (Fig. 4E, 4F, 4I, 4J), consistent with the enhanced pSmad1/5/8 activity in this domain. In the posterior palate, ectopic expression of *Shox2* and *Msx1* was detected in the mesenchyme of mutant embryos, coinciding with the area where ectopic pSmad1/5/8 positive cells were observed (Fig. 4G, 4H, 4K, 4L).

**Figure 5:**
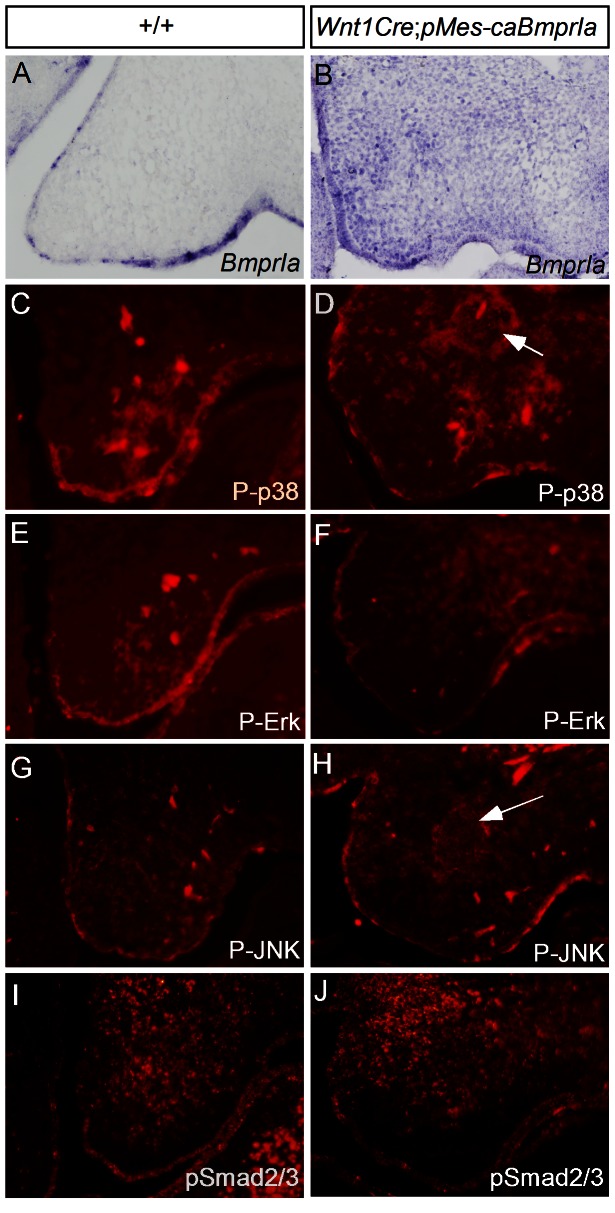
Ectopic activation of BMP non-canonical signaling pathways in *Wnt1Cre*;*pMes*-*caBmprIa* palatal shelves. (A, B) In situ hybridization shows ectopic expression of *BmprIa* in the palatal mesenchyme of E13.5 transgenic embryo (B), compared to *BmprIa* expression in wild type littermate (A). (C–H) Immunohistochemical staining shows expression of activated BMP non-canonical signaling mediators in E13.5 control and transgenic palatal shelves. Note ectopic expression (arrows) of P-p38 (D) and P-JNK (H) in the transgenic palatal mesenchyme. (I, J) Immunohistochemical staining shows expression of pSmad2/3 in E13.5 control (I) and transgenic palatal shelves (J).

Since pSmad1/5/8 were not uniformly activated in the palatal mesenchymal cells of *Wnt1Cre*;*pMes-caBmprIa* mice, we wondered if this is attributed to selective expression of the *caBmprIa* transgenic gene. We examined *caBmprIa* expression in the transgenic palatal mesenchyme by in situ hybridization. We selected the palatal region at the first molar level where endogenous *BmprIa* is only expressed in the palatal epithelium (Fig. 5A; 13). As shown in Fig. 5B, *caBmprIa* transcripts were detected uniformly in the palatal mesenchyme. We further determined if expression of *caBmprIa* could alter the activity of TGFβ/BMP non-canonical signaling pathways by examining the expression of P-p38, P-Erk, and P-JNK. As shown in Fig. 5, the expression of these non-canonical TGFβ/BMP signaling pathways was not enhanced in general. However, similar to pSmad1/5/8 expression, an ectopic mass of P-p38 and P-JNK positive cells was also detected (Fig. 5D, 5H). In addition, we did not see a change in pSmad2/3 expression in the transgenic palate, as compared to wild type control (Fig. 5I, 5J). These observations suggest that selective groups of palatal mesenchymal cells respond activation of BMPRIa-mediated signaling.

**Figure 6:**
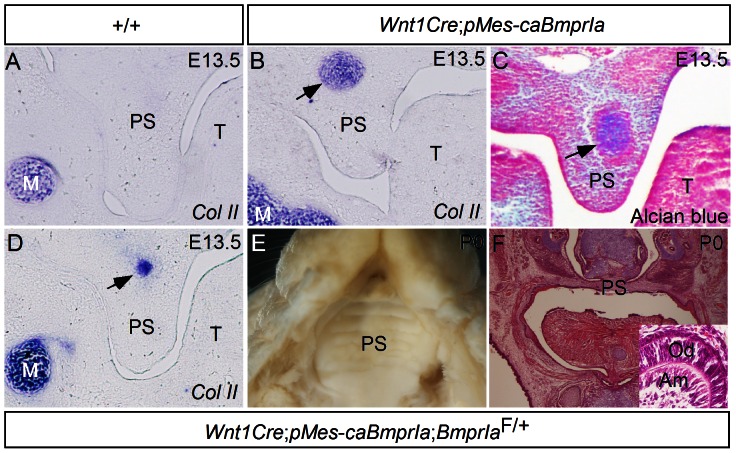
Enhanced BMP signaling induces ectopic cartilage formation in the palatal shelves. (A) In situ hybridization detects *Col II* expression in the Meckel's cartilage but not in the palatal shelf of an E13.5 wild type embryo. In situ hybridization shows an ectopic *Col II*-positive domain (arrow) within the palatal shelf of an E13.5 *Wnt1Cre*;*pMes*-*caBmprIa* embryo. (C) Alcian blue staining shows presence of an ectopic cartilage (arrow) within the palatal shelf of an E13.5 *Wnt1Cre*;*pMes*-*caBmprIa* embryo. (D) In situ hybridization shows a small ectopic *Col II*-positive cell mass (arrow) in the palatal shelf of an E13.5 *Wnt1Cre*;*pMes*-*caBmprIa*;*BmprIa* ^F/+^ embryo. (E, F) Whole mount and section of P0 *Wnt1Cre*;*pMes*-*caBmprIa*;*BmprIa* ^F/+^ mice show normal palate formation. Insert in (F) shows well differentiated ameloblasts and odontoblasts. T, tongue; Am, ameloblasts; Od, odontoblasts; PS, palatal shelf.

Histological analysis revealed formation of enlarged and ectopic cartilages in craniofacial region of *Wnt1Cre*;*pMes-caBmprIa* mice (Fig. 1F, 1H). Since an ectopic condensed mesenchymal cell mass was observed in the posterior domain of each palatal shelf of E13.5 transgenic embryo (Fig. 2D) where ectopic pSmad1/5/8, P-p38, and P-JNK positive cells and expression of *Shox2* and *Msx1* were detected (Fig. 4; 5), we wondered if this condensed cell mass represents a condensation of precartilagious cells and the formation of ectopic cartilage within the palatal shelves could contribute to deformed palate morphology and subsequently to the cleft palate defect. We examined in the developing palatal shelves the expression of type II collagen (*Col II*), a molecular marker for proliferating cartilage cells. No *Col II* expression was detected in the palatal shelves of E13.5 control embryo (Fig. 6A). However, ectopic *Col II* expression domain was indeed found in the posterior palatal shelves of mutant embryos, overlapping with the area where ectopic pSmad1/5/8, P-p38, and P-JNK positive cells and expression of *Shox2* and *Msx1* were observed (Fig. 6B). The presence of ectopic cartilage was further confirmed by Alcian Blue staining (Fig. 6C). All 9 samples of E13.5 mutants that were subjected to in situ hybridization for *Col II* and Alcian Blue staining presented ectopic cartilages in the developing palatal shelves. To determine if the ectopic cartilage formation in the posterior palatal mesenchyme may contribute to the cleft palate formation in *Wnt1Cre*;*pMes-caBmprIa* mice, we crossed a floxed *BmprIa* allele onto the *Wnt1Cre*;*pMes*-*caBmprIa* background. While formation of an ectopic cartilage was still found in the posterior palatal shelf of E13.5 *Wnt1Cre*;*pMes*-*caBmprIa*;*BmprIa* ^F/+^ mice, the size of the cartilage was dramatically reduced as compared to that found in *Wnt1Cre*;*pMes*-*caBmprIa* palate (Fig. 6D). Under such *BmprIa* haploinsuficient background, not just the size of ectopic cartilage was reduced, but the cleft palate defect was also completed rescued in *Wnt1Cre*;*pMes-caBmprIa* mutants (N = 5; Fig. 6E, 6F). In addition, *Wnt1Cre*;*pMes*-*caBmprIa*;*BmprIa* ^F/+^ mice also exhibited fairly differentiated odontoblasts and ameloblasts, as assessed by their well elongated morphology (Insert in Fig. 6F). These results suggest that the ectopic cartilage formed in the palatal shelves could represent one causative for the cleft palate defect in *Wnt1Cre*;*pMes-caBmprIa* mutants and further support a requirement for finely regulated BMPRIa-mediated signaling in normal palate development.

### Delayed odontogenic differentiation in Wnt1Cre;pMes-caBmprIa mice

**Figure 7:**
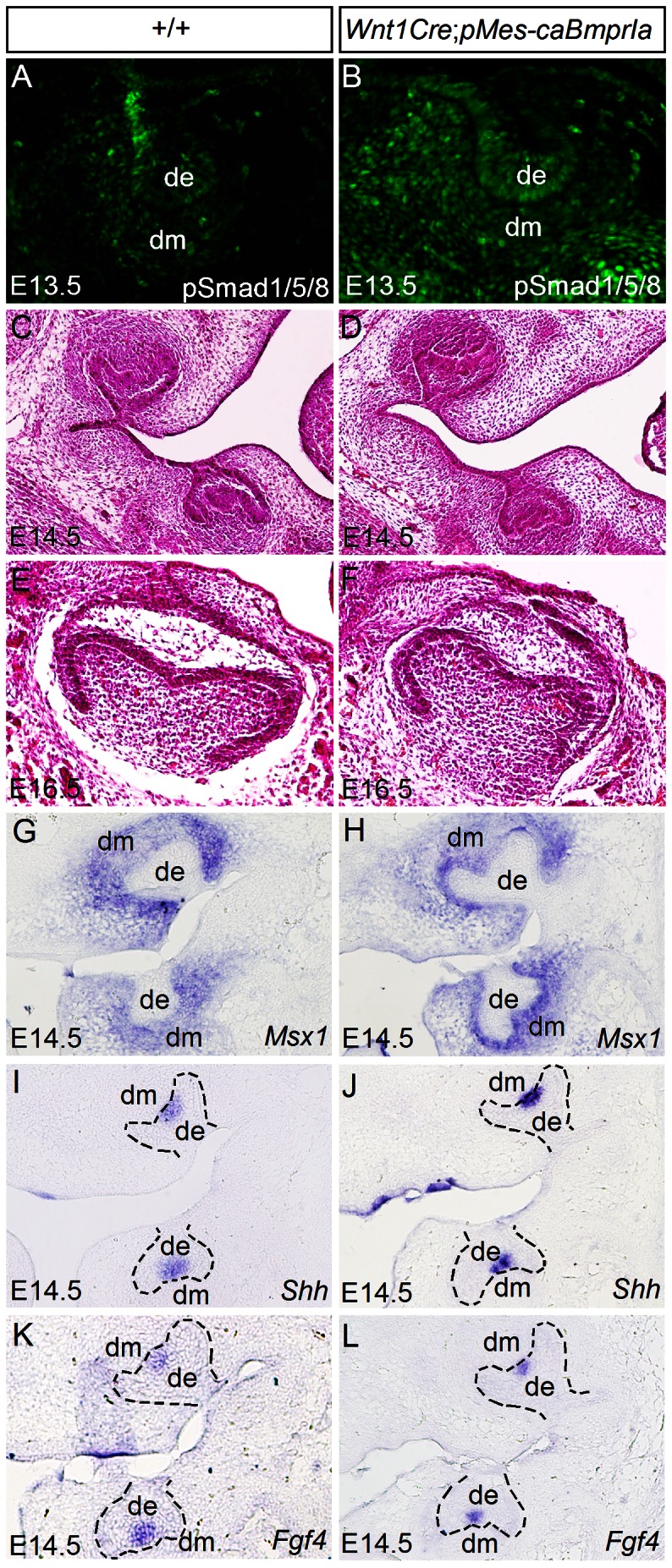
Unaffected early molar development and gene expression in *Wnt1Cre*;*pMes* **-** *caBmprIa* mice. (A, B) Immunostaining shows enhanced pSmad1/5/8 signals in the molar germ of E13.5 *Wnt1Cre*;*pMes*-*caBmprIa* embrys (B) as compared to the control (A). (C–F) Coronal sections show comparable molar structures of E14.5 (C D) and E16.5 (E, F) wild type (C, E) and transgenic embryos (D, F). (G–L) In situ hybridization shows comparable expression levels and patterns of *Msx1* (G, H), *Shh* (I, J) and *Fgf4* (K, L) in the molars of E14.5 controls (G, I, K) and transgenic embryos (H, J, L). de, dental epithelium; dm, dental mesenchyme.

Since histological analyses revealed a less differentiated status of odontoblasts and ameloblasts as well as lack of dentin deposition in *Wnt1Cre*;*pMes*-*caBmprIa* molars at P0 (Fig. 1L), we wondered if this delayed odontogenic differentiation is caused by early developmental defects and altered gene expression. We conducted histological analyses on early molar development and examined the expression of a few genes known to be important for tooth development and patterning. We first confirmed that the expression of *caBmprIa* in CNC lineage indeed leads to overactive BMP signaling in the dental mesenchyme by immunohistochemical staining on the expression of pSmad1/5/8. The number of pSmad1/5/8 positive cells was indeed significantly increased in the dental mesenchyme of the *Wnt1Cre*;*pMes-caBmprIa* molar (Fig. 7A, 7B). Histological examinations manifested comparable molar structures between controls and transgenic animals at the E14.5 cap and the E16.5 bell stages (Fig. 7C–F). Consistent with normal tooth development, the expression of *Msx1* in the dental mesenchyme and the expression of *Shh* and *Fgf4* in the enamel knot of the transgenic molar at E14.5 remained at the levels and in the patterns identical to that observed in the controls (Fig. 7G–L). These results indicated the early tooth development was not affected in *Wnt1Cre*;*pMes-caBmprIa* mice.

**Figure 8:**
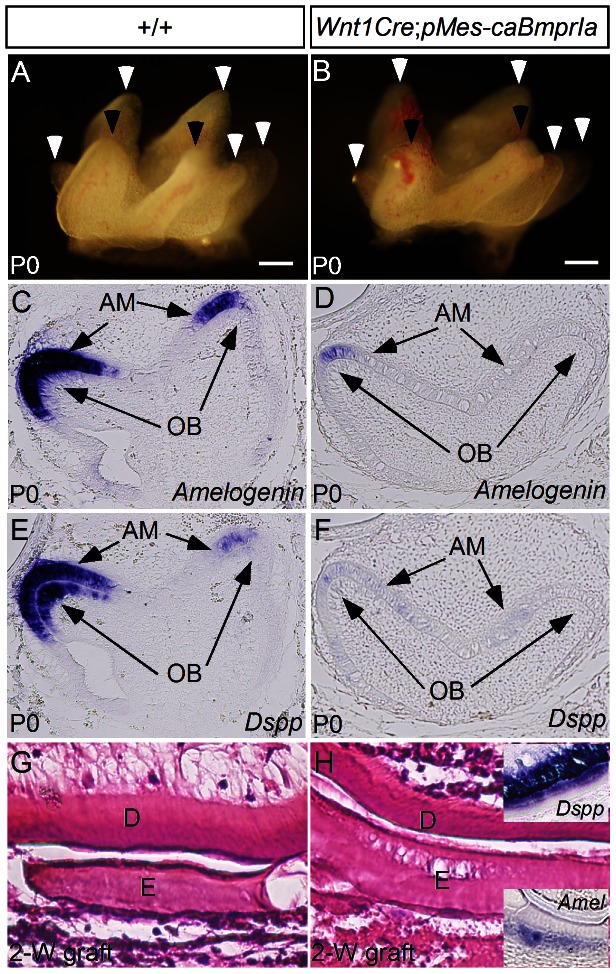
Enhanced BMP signaling activity does not affect size and cusp patterning but delays odontogenic differentiation. (A, B) Whole mount images of P0 wild type (A) and *Wnt1Cre*;*pMes*-*caBmprIa* (B) molar shows comparable size and cusp patterns. (C–F) In situ hybridization shows strong expression of *Amelogenin* and *Dspp* in P0 wild type molar (C, E), but barely detectable expression of these two genes in P0 transgenic molar (D, F). (G, H) histological analyses show deposition of dentin and enamel in tooth grafts of wild type control (G) and *Wnt1Cre*;*pMes*-*caBmprIa* (H) molar after 2 weeks in kidney capsule culture. Inserts in (H) show *Dspp* and *Amelogenin* expression in the transgenic grafts. D, dentin; E, enamel; AM, ameloblasts, OB, odontoblasts. Scale bar = 200 µm.

Despite normal early development and normal size and patterning of the molars at P0 (Fig. 8A, 8B), examination of the expression of odontogenic differentiation markers revealed a delayed differentiation of both ameloblasts and odontoblasts, as assessed by barely detectable expression of *Amelogenin* and *Dspp*, the molecular markers for differentiated/differentiating ameloblasts and odontoblasts, respectively, in the P0 transgenic molars, whereas strong expression of these two genes was detected in the controls at the same age (Fig. 8C–F). To determine if the lower level of *Dspp* and *Amelogenin* expression in the teeth of *Wnt1Cre*;*pMes-caBmprIa* mice represents either a delayed or an arrested odontogenic differentiation, we grafted mandibular molars from E13.5 *Wnt1Cre*;*pMes-caBmprIa* embryos and wild type controls underneath mouse kidney capsule. After 2 weeks in subrenal culture, transgenic grafts, similar to the controls, formed teeth with deposition of dentin and enamel and expression of *Amelogenin* and *Dspp* (N = 7; Fig. 8G, 8H), indicating that overly activated BMP signaling in the dental mesenchyme causes delayed but not arrested differentiation of odontoblasts and ameloblasts.

## Discussion

The essential role for BMP signaling in the development of craniofacial organs including the palate and tooth has been studied extensively using loss-of-function approach. We have shown previously that BMP signaling homeostasis is equally importance for tooth and palate development, as evidenced by the formation of cleft palate in mice carrying transgenic expression of *caBmprIa* in the epithelium as well as the defective palate development and absence of upper incisors in mice lacking the BMP antagonist Noggin [11], [13], [36]. In this study, we present additional evidence for the requirement of finely tuned BMP activity in the mesenchymal component for normal palate and tooth development. We show that enhanced BMPRIa-mediated signaling in the CNC lineage leads to complete clefting of the secondary palate and delayed odontogenic differentiation in addition to the formation of ectopic cartilages in the craniofacial region. It was also shown recently that elevated BMPRIa-mediated BMP signaling in CNCs causes craniosynostosis in mice [37].

In the developing palatal shelves, *BmprIa* is expressed in both the epithelium and mesenchyme of the anterior palate, but is expressed only in the epithelium of the posterior region [13]. Consistent with this expression pattern is that mesenchymal inactivation of *BmprIa* results in defective cell proliferation in the anterior palatal mesenchyme and subsequent formation of a unique anterior clefting of the secondary palate [13]. Interestingly, in our current *caBmprIa* overexpression model, similar defective cell proliferation was also found in the anterior palatal mesenchyme but not in the posterior palate. These phenotypes were also observed in the *Noggin* mutant palate in which cell proliferation rate is reduced in the anterior palatal mesenchyme, but is remained unaffected in the posterior palatal mesenchyme [11]. These differential cellular responses in terms of cell proliferation to elevated BMP signaling in the mesenchymal tissue along the anterior-posterior axis of the palatal shelf is consistent with our previous findings that exogenously applied BMP2 or BMP4 induces cell proliferation in the anterior but not in the posterior palatal mesenchyme [9], [10]. Several other signaling pathways have also been implicated in cell proliferation regulation in the developing palate, including FGF, TGF-β, and Wnt [5], [38], [39], [40], [41], [42], [43]. The observations that either reduced or elevated BMP signaling leads to a decreased level of cell proliferation in the anterior palatal mesenchyme indicate that a precisely controlled homeostasis of BMP signaling activity is a critical component of the regulatory signaling network that controls cell proliferation. Alteration in BMP signaling homeostasis could interrupt the balance of the regulatory signaling network, causing aberrant cell proliferation.

While deletion of *BmprIa* in the palatal mesenchyme caused dramatic down-regulation of BMP responsive genes including *Msx1* and *Shox2* [13]), augmentation of BMPRIa-mediated signaling did not enhance the expression levels of *Msx1* and *Shox2* in the anterior palatal mesenchyme. Instead, this enhanced BMP signaling induced ectopic expression of these two genes in the posterior palatal mesenchyme. However, it was shown previously that application of exogenous BMP2 or BMP4 failed to induce expression of *Msx1* and *Shox2* in the posterior palatal mesenchyme [9], [10], [35]. The lack of *BmprIa* and *BmprIb* expression in the posterior palatal mesenchyme could explain failed response of the palatal mesenchyme in terms of gene expression to exogenous applied BMP induction [13]. The restricted ectopic domain of Smad1/5/8 phosphorylation along with activation of BMP non-canonical signaling regulators p38 and JNK and *Msx1* and *Shox2* expression in the posterior palate of *Wnt1Cre*;*pMes*-*caBmprIa* mice indicates a selective response of CNC-derived cells to BMP signaling. This ectopic expression of BMP canonical and non-canonical mediators (pSmad1/5/8, P-p38, P-JNK) and *Msx1* and *Shox2* appears to be responsible for the formation of ectopic cartilage in the posterior palatal shelf. The presence of ectopic cartilage seems to cause a deformed posterior palate structure (shorter and wider compared to control) and delayed palate elevation. This idea is supported by the correlation of the presence of an ectopic cartilage with dramatically reduced size in the palatal shelf and subsequent formation of an intact palate in *Wnt1Cre*;*pMes*-*caBmprIa* mice on a *BmprIa* haploinsufficient background. Nevertheless, these observations further confirm an absolute requirement of BMP signaling homeostasis in CNC-derived tissue for palate development.

Despite an elevated level of pSmad1/5/8 in the developing tooth germ in *Wnt1Cre*;*pMes*-*caBmprIa* mice, early tooth development, gene expression as well as cusp patterning appeared normal. However, the differentiation of odontoblasts and ameloblasts was delayed. These observations indicates that enhanced BMP signaling in the dental mesenchyme does not exert a detrimental effect on early tooth development and patterning, suggest that the developing tooth has a higher tolerance to overactive BMP signaling compared to the developing palatal shelves. This notion is consistent with phenotypes observed in *Noggin* mutant mice, an alternative gain-of BMP signaling function model, in which a cleft palate formed, but the molars and lower incisors developed normally except an early fusion of upper incisors [11], [36], [44], [45]. However, enhanced BMP activity in the dental mesenchyme has an effect at the late developmental stage, causing delayed odontogenic differentiation. Many studies have implicated a role of BMP signaling in the differentiation of odontoblasts and ameloblasts, as evidenced by the expression of multiple *Bmp* genes in the differentiating/differentiated odontoblasts and ameloblasts [46]. The facts that BMPs are able to induce odontoblasts to produce dentin and the lack of Smad4 prevents terminal odontoblast differentiation, as well as that overexpression of *Follistatin*, a BMP inhibitor, inhibits ameloblast differentiation support a positive role for BMP signaling in promoting odontogenic differentiation [47], [48], [49], [50]. However, in our transgenic model, overactive BMP signaling appears to exert an opposite role in odontogenic differentiation. Several other signaling pathways are also involved in the regulation of odontogenic differentiation, including TGFβ, Shh, and Wnt, forming a complicated regulatory network [51]. While the mechanism underlying the delayed odontogenic differentiation in *Wnt1Cre*;*pMes*-*caBmprIa* mice is currently unknown, and warrants future investigation, the enhanced BMP signaling in the dental mesenchymal component may disrupt the balance of this tightly regulated signaling network, leading to a delayed differentiation. Since *caBmprIa* is forced to be expressed in the dental mesenchymal cells but not in the dental epithelial cells and the differentiation of ameloblasts relies on instructive signals from the differentiating/differentiated odontoblasts and predentin, the delayed ameloblast differentiation is a non-cell autonomous effect and a secondary consequence of aberrant signaling network in the odontoblasts.

In conclusion, our studies using a gain-of-function approach reveal the importance of homeostasis of BMPRIa-mediated signaling in CNC-derived tissue component in palate and tooth development. Augmented BMP signaling leads to cleft palate formation and delayed odontogenic differentiation.
